# Rapidly Progressing Retropharyngeal Abscess in a Six-Month-Old: Anesthetic and Diagnostic Challenges Leading to Lemierre’s Syndrome

**DOI:** 10.7759/cureus.90187

**Published:** 2025-08-15

**Authors:** Ashley Walker, Nicholas Lugo, Hammza Elaref, Qaswara Elaref, Sharif Mohamed

**Affiliations:** 1 Anesthesiology, University of Texas Medical Branch, Galveston, USA; 2 Anesthesiology, California State Polytechnic University, Pomona, USA

**Keywords:** direct laryngoscopy (dl), lemierre's syn, pediatric-anesthesia, retropharyngeal abscesses, tonsillitis

## Abstract

Retropharyngeal abscesses (RPAs) are serious infections in children that often stem from tonsillitis and can quickly lead to life-threatening complications due to the unique anatomy of the neck. These abscesses commonly present with fever, difficulty swallowing, and respiratory distress, and can progress to conditions such as mediastinitis, empyema, and Lemierre’s syndrome (LS) if left untreated. This case report follows a six-month-old infant who initially presented with persistent fever, anasarca, and a suspected deep neck space infection. The RPA rapidly worsened, resulting in empyema, LS, and a pseudoaneurysm of the internal carotid artery. The patient required multiple surgeries, including drainage of abscesses in the neck and chest, as well as treatment for thromboembolic and respiratory complications. This case underscores the importance of early diagnosis, quick intervention, and careful anesthetic management in preventing severe outcomes in children with RPAs.

## Introduction

Retropharyngeal abscesses (RPAs) are deep neck space infections involving the space located posterior to the pharynx and anterior to the prevertebral fascia [1]. These infections most commonly occur in children under five years of age due to the presence of retropharyngeal lymph nodes, which typically atrophy with age [2]. In infants, diagnosis is particularly challenging because their nonspecific symptoms, such as fever, irritability, poor feeding, or abdominal distension, often resemble those of more common pediatric illnesses. A nationwide population-based study reported an annual incidence of 2.64 cases per 100,000 individuals, with a higher prevalence among young males [3]. 

Clinically, patients often present with fever, neck stiffness, dysphagia, drooling, and respiratory distress; stridor may develop in more advanced cases. If not promptly recognized and treated, RPAs can progress rapidly and result in life-threatening complications [1,2]. One of the most dangerous routes of spread is through the "danger space," a potential fascial plane that extends from the skull base to the diaphragm, allowing direct communication with the mediastinum [4]. This can precipitate mediastinitis, pleural involvement, and empyema, each of which poses a significant threat to respiratory function and necessitates urgent surgical and medical intervention [5].

Delayed diagnosis or inadequate management can lead to catastrophic outcomes, including airway obstruction, sepsis, multiorgan failure, and death [6]. Furthermore, extension of infection to the internal jugular vein may result in septic thrombophlebitis and the development of Lemierre’s syndrome (LS), a rare but severe complication marked by septic emboli and systemic sepsis that typically follows an oropharyngeal infection such as pharyngitis or tonsillitis. This can result in the spread of abscesses to various organs, most commonly the lungs [7]. 

This case report describes a pediatric patient with an extensive RPA secondary to tonsillitis, complicated by mediastinitis, bilateral empyema, and LS. It highlights the critical importance of early recognition and aggressive management of deep neck infections to prevent progression to life-threatening thoracic and systemic complications.

## Case presentation

A previously healthy six-month-old male, born at term via an uncomplicated delivery except for maternal gestational diabetes, up to date on immunizations and meeting developmental milestones, was transferred from an outside hospital to the pediatric intensive care unit (PICU) with persistent fever, anasarca, and a suspected deep neck space infection. On admission, the patient was critically ill with hypoxemic respiratory failure, bilateral pleural effusions, ascites, hepatosplenomegaly, and generalized edema. Respiratory support included a high-flow nasal cannula at 45% FiO₂ and 8 L/min. Laboratory evaluation revealed leukocytosis with bandemia, microcytic anemia, coagulopathy, hypophosphatemia, and hypoalbuminemia (Table 1). A positive COVID IgG indicated prior infection. Given persistent fever, multiorgan involvement, and elevated inflammatory markers, the patient met diagnostic criteria for multisystem inflammatory syndrome in children (MIS-C) and was started on intravenous immunoglobulin (IVIG), methylprednisolone, and aspirin.

**Table 1:** Investigations done on the day of admission to the UTMB PICU WBC Count: white blood cell count; BANDS: band neutrophils (immature white blood cells), values and units were not provided; MCV: mean corpuscular volume; PT INR: prothrombin time international normalized ratio; aPTT: activated partial thromboplastin time; CRP: C-reactive protein

Investigation	Value (Day of Admission)	Reference Range
WBC Count (x10^3^/µL)	25.49	6.00-17.50
BANDS	Increased	N/A
Hemoglobin (g/dL)	7.8	9.5-13.5
Hematocrit (%)	24.1	29.0 - 41.0
MCV (fL)	78.8	72.0-82.0
PT INR	1.5	<1.1
aPTT (seconds)	32	26-36
Fibrinogen (mg/dL)	261	167-453
Platelet Count (x10^3^/µL)	543	133-320
Phosphorus (mg/dL)	4.1	4.5-6.7
Albumin (g/dL)	2.9	3.5-5.0
CRP (mg/dL)	17.5	<0.8
Lactic Acid (mmol/L)	1.03	0.50-2.20

**Figure 1:**
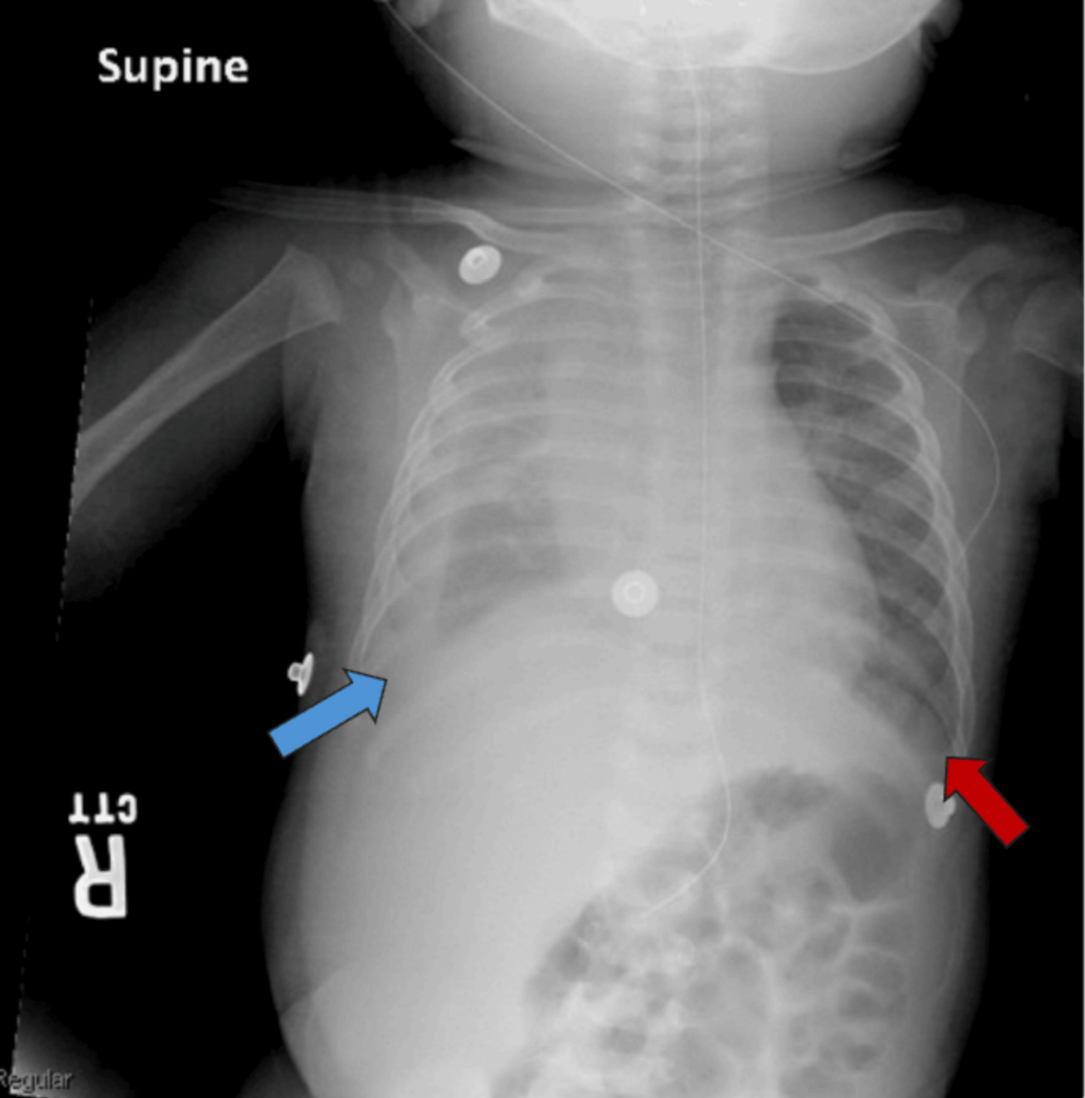
Frontal chest radiograph demonstrating a right-sided moderate pleural effusion (blue arrow) and a left-sided small-to-moderate pleural effusion (red arrow). No evidence of pneumothorax or acute osseous abnormalities.

Initial imaging included an unremarkable head CT and a chest radiograph, which showed bilateral pleural effusions (Figure 1). Lumbar puncture was deferred due to oxygen desaturation. An echocardiogram showed no significant cardiac abnormality. Given the patient's clinical presentation, indications for endotracheal intubation included airway protection, respiratory distress, and impending respiratory failure. The patient was maintaining spontaneous ventilation at the time of intervention. Following sedation with dexmedetomidine 0.30 mcg/kg/hr, fentanyl 2.00 mcg/kg/hr, midazolam 0.10 mg/kg/hr, and vecuronium 1.00 mcg/kg/hr, endotracheal intubation was successfully performed after a single attempt. The patient was preoxygenated and maintained spontaneous ventilation under deep sedation prior to airway instrumentation. Manual in-line stabilization was maintained throughout. Intubation was performed orally using video laryngoscopy with a Miller #1 blade and an intubating stylet, yielding a Cormack-Lehane Grade I view. A 3.5 mm cuffed endotracheal tube was placed to a depth of 12 cm at the teeth, with position confirmed by chest auscultation.

**Figure 2:**
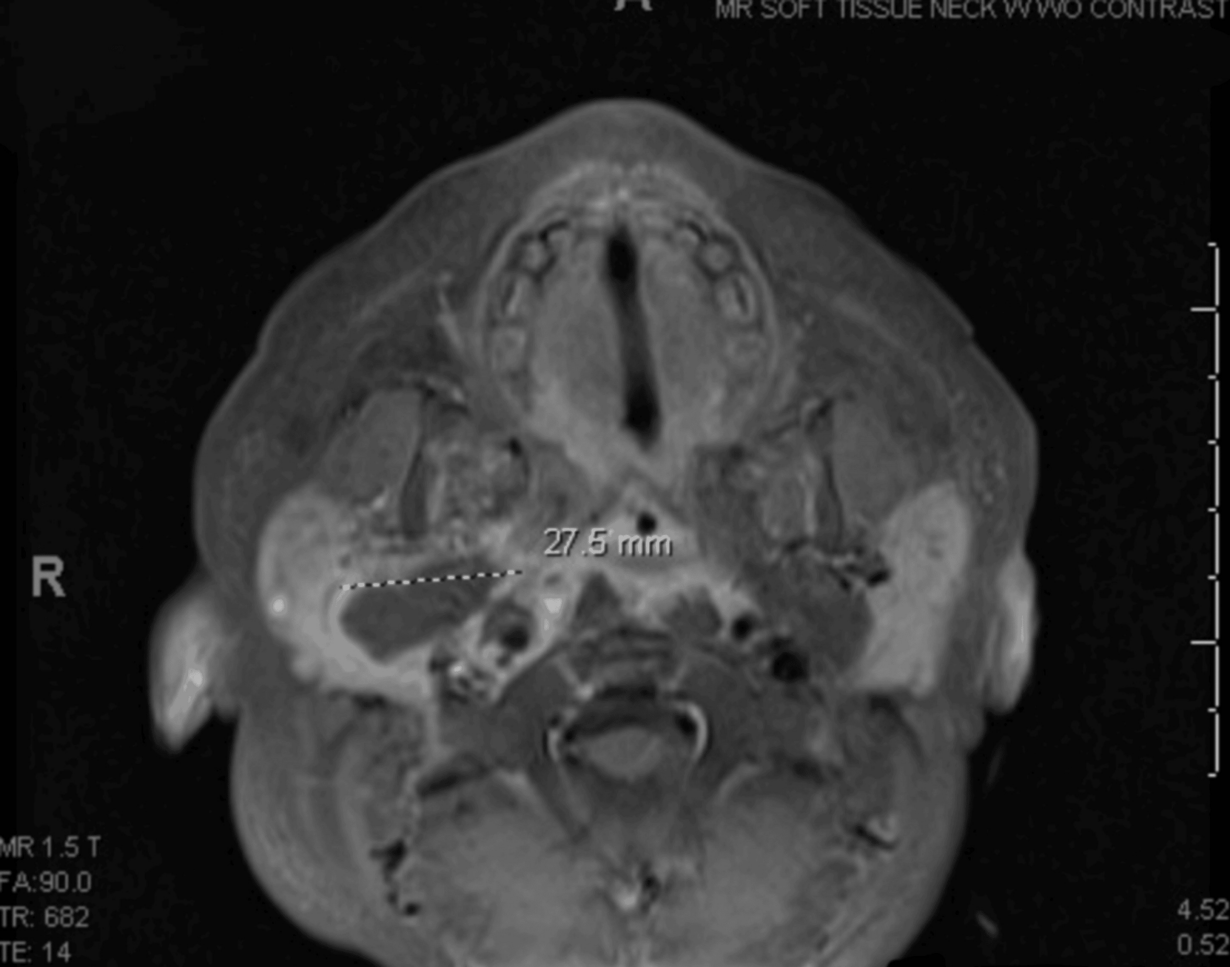
Axial post-contrast MRI of the neck demonstrating a 27.5 mm rim-enhancing fluid collection in the right parapharyngeal space, consistent with a deep neck space abscess. The collection involves the carotid and retropharyngeal spaces, with associated soft tissue edema and cellulitis.

**Figure 3:**
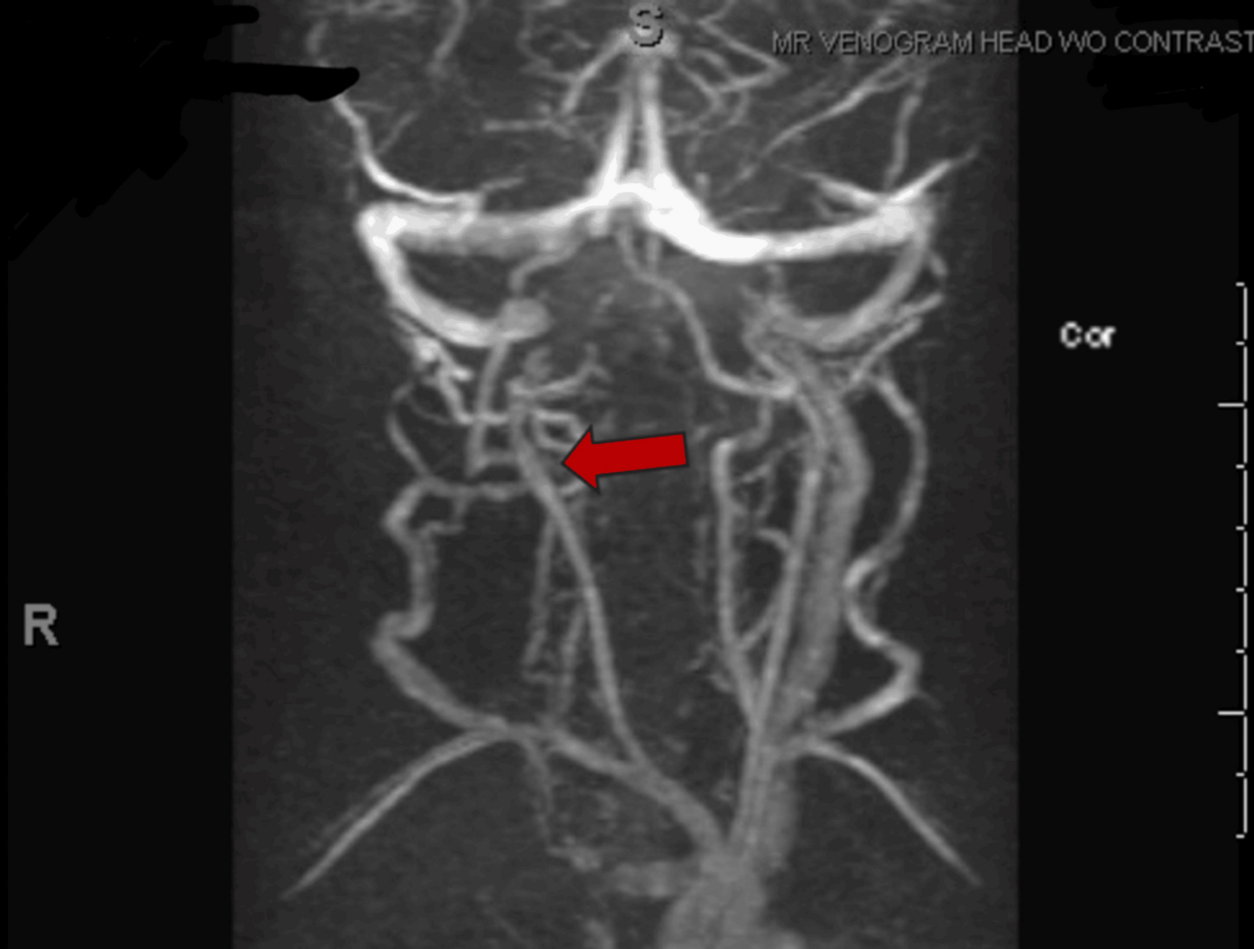
Time-of-flight MR venogram of the head and neck demonstrating absent flow in the right internal jugular vein (red arrow), consistent with venous thrombosis secondary to adjacent deep neck infection.

MRI (Figure 2) and venogram of the neck (Figure 3) confirmed multiple abscesses involving the retropharyngeal, carotid, and parapharyngeal spaces, with communication to mediastinal loculated fluid collections. Thrombosis of the right internal jugular vein was identified, consistent with LS. Surgical intervention included incision and drainage of the neck and mediastinal abscesses, placement of a Jackson-Pratt (JP) drain and bilateral chest tubes, placement of a central venous access catheter in the right femoral vein, and bronchoscopy. Intraoperative findings revealed caseous necrosis within the carotid sheath and purulent drainage. Cultures grew methicillin-sensitive Staphylococcus aureus (MSSA).

**Figure 4:**
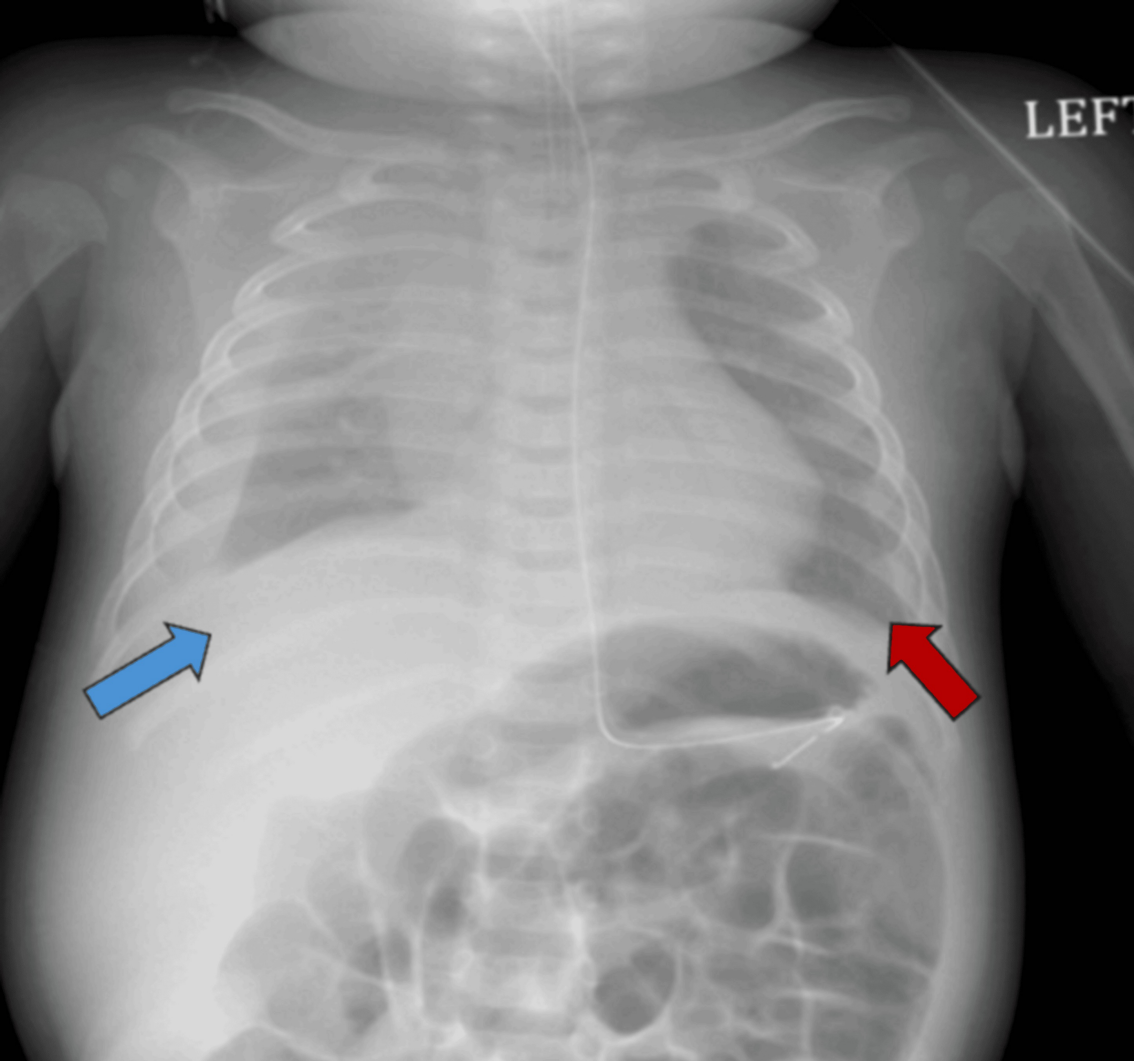
Frontal chest radiograph demonstrating a moderate-to-large right pleural effusion (blue arrow), with interval improvement compared to the prior study. A small, stable left pleural effusion is also present (red arrow). Diffuse hazy opacities are noted throughout the bilateral lung fields, without significant interval change.

The patient remained intubated with ongoing sedation and paralysis. Over the following days, he developed bilateral empyema requiring repeated chest tube drainage procedures and intrapleural fibrinolytic therapy. Imaging demonstrated persistent mediastinal abscesses, bilateral pleural effusions (Figure 4), and evolving pulmonary complications, including pneumonia and probable pulmonary abscesses. Subsequent CT and MRI later revealed a pseudoaneurysm of the right internal carotid artery and suspected thrombus propagation into the right sigmoid sinus and jugular bulb.

Additional procedures included flexible bronchoscopy, direct laryngoscopy with intraoral drainage, myringotomy with tympanostomy tube placement, and removal of otowick packing. Alteplase was given intrapleurally to address loculated fluid collections. The patient received multiple transfusions for anemia secondary to surgical blood loss and procedural bleeding. Fluconazole was initiated for suspected fungal colonization. Aspirin was continued to manage the internal carotid artery aneurysm.

Throughout hospitalization, the patient remained intubated and mechanically ventilated. The patient was empirically started on triple antibiotic therapy consisting of linezolid, ceftriaxone, and metronidazole to provide broad coverage for Gram-positive, Gram-negative, and anaerobic organisms, given the extensive deep neck space infection, mediastinal extension, and bilateral empyemas. This regimen was maintained while intubated to prevent nosocomial pneumonia and to ensure coverage for polymicrobial pathogens, including Fusobacterium necrophorum in the context of Lemierre’s syndrome. Anticoagulation was withheld due to surgical considerations. Sedation was titrated for airway protection and comfort. By hospital day 14, he remained intubated with stable hemodynamics and improving laboratory parameters, including hemoglobin and platelet counts. All drains had been removed, except the endotracheal tube. Sedation was increased slightly in preparation for transfer.

He was subsequently accepted for transfer to a tertiary care facility for continued management of the internal carotid artery pseudoaneurysm and airway care. At the time of transfer, the patient remained intubated but hemodynamically stable, with improving inflammatory markers and a comprehensive medical and surgical plan established at the receiving institution.

At the external facility, the patient underwent intravascular coiling of the right internal carotid artery by neurosurgery, followed by incision and drainage of an RPA performed by ENT. Nineteen days after his initial intubation, he was successfully extubated to high-flow nasal cannula and gradually weaned to room air. Throughout his hospitalization, he remained on intravenous antibiotics, and imaging was used to monitor his progress.

Initial head CTs showed bilateral subdural hygromas, persistent otomastoiditis, a small right frontal extra-axial hematoma, and a small thrombus in the right sigmoid sinus. Chest imaging revealed progression of mediastinal infection with rim-enhancing abscesses extending along the right jugular vein and compressing central vasculature, in addition to a right pleural effusion and airspace disease, both of which showed improvement on subsequent scans.

A laryngeal ultrasound performed at the tertiary facility showed normal vocal cord movement without evidence of paralysis. A videofluoroscopic swallow study (VFSS), also completed externally, indicated deep laryngeal vestibular penetration with all consistencies and silent aspiration of thin liquids. These interpretations were based on radiology reports; original imaging was not available for review. In response, feeding strategies were adjusted in consultation with speech-language pathology.

As sedation was weaned, he experienced intermittent withdrawal symptoms and transient hypertension, both of which were treated with clonidine. He also received intermittent doses of Lasix for anasarca. His nutritional intake was initially poor, requiring nasogastric feeding. With the help of speech and occupational therapy, he gradually transitioned to full oral feeds. Although his weight initially declined, it began to trend upward prior to discharge.

**Figure 5:**
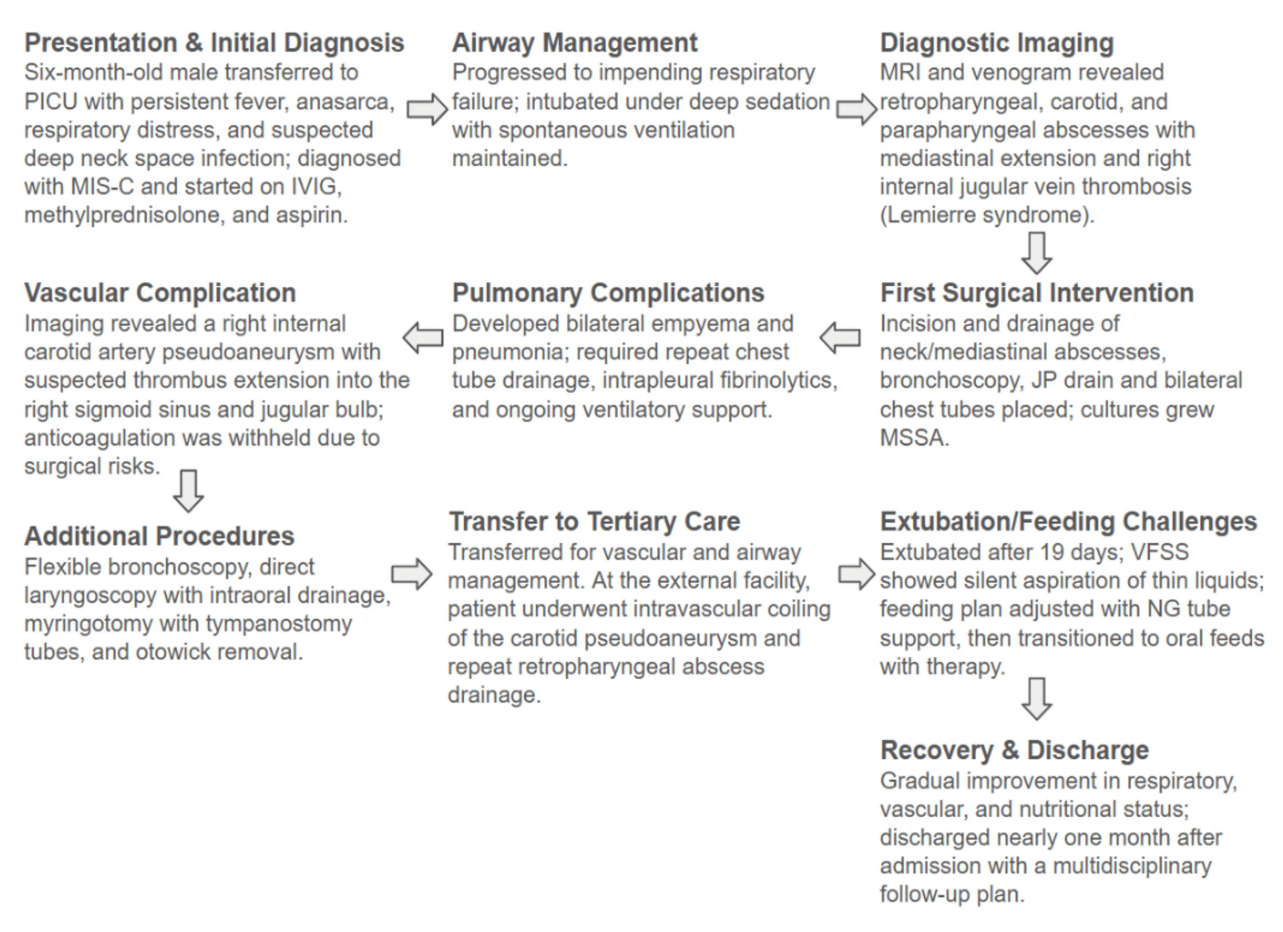
A visual breaking down the case presentation into ten main clinical and management milestones in chronological order. PICU: pediatric intensive care unit, MIS-C: multisystem inflammatory syndrome in children, IVIG: intravenous immunoglobulin, JP drain: Jackson-Pratt drain, MSSA: methicillin-sensitive Staphylococcus aureus, VFSS: videofluoroscopic swallow study, NG: nasogastric

By the time of discharge, nearly one month after admission, he was tolerating feeds well and in stable condition. A comprehensive discharge plan was put in place, including follow-up with multiple specialties and thorough caregiver education regarding medications, return precautions, and care coordination. A summary of the case presentation can be found in Figure 5.

## Discussion

This case report presents a severe presentation of an RPA in a six-month-old infant, complicated by LS, bilateral empyemas, and an internal carotid artery pseudoaneurysm. The patient’s age, involvement of the airway, and the extent of the infection made this case particularly challenging. Children often have delayed clinical presentations, and this danger can be exacerbated in patients such as infants who lack proper communication skills [8]. RPAs in young children are typically managed with antibiotics and drainage; however, in more advanced cases such as this one, they can quickly become life-threatening. For anesthesiologists in particular, these infections raise serious concerns due to the potential for rapid airway deterioration and difficult intubation. Given the significant risk of airway obstruction, a well-planned approach to airway management is essential, often requiring intubation in a controlled environment such as the operating room. This is particularly true in pediatric patients, where smaller airways and developmental differences increase the complexity of care. Anesthesiologists play a key role in maintaining airway safety and patient stability throughout the process [9]. Coordinated care among otolaryngologists, radiologists, and anesthesiologists is also crucial to achieving the best possible outcomes [10].

RPAs can distort the airway through swelling, inflammation, and mass effect. In this case, imaging revealed that the abscess had spread into the parapharyngeal, carotid, and mediastinal spaces, raising concerns about airway obstruction and the risk of rupturing the abscess during intubation. Intubating an infant with an RPA is especially challenging because of the small size of their airway and the easy collapsibility of the airway if spontaneous breathing effort and muscle tone are lost, necessitating maintaining the spontaneous breathing effort during airway management. Manipulating the infected tissue can easily lead to rupture or aspiration, further complicating the situation. Advanced airway management techniques, such as video-laryngoscopy or awake fiberoptic intubation, are often required for enhanced visualization to avoid airway trauma while maintaining spontaneous ventilation. In this case, video laryngoscopy was utilized to secure the airway, and intubation was successfully performed on the first attempt following adequate sedation. This approach allowed for careful airway visualization while minimizing manipulation of inflamed tissues and reducing the risk of abscess rupture [11].

The retropharyngeal space directly communicates with the parapharyngeal space and lies just anterior to the danger space, creating a pathway for the rapid spread of infection. These anatomical features allow it to extend into the mediastinum and carotid sheath, leading to serious complications such as mediastinitis, empyema, and LS. Mediastinitis is characterized by inflammation or infection of structures such as the heart, major blood vessels, esophagus, trachea, and lymph nodes. Previous studies have suggested mediastinitis is more closely linked to RPAs than other deep neck infections [12]. Management often includes supportive care, broad-spectrum antibiotics, and surgical drainage. Chest imaging typically reveals mediastinal widening and bilateral pleural effusions, as seen in our case, and highlights the benefits of frequent chest imaging in such cases. The proximity of the mediastinum to the lungs allows for further complications, such as an empyema, to develop. Empyemas can lead to severe respiratory insufficiencies that require constant intubation with mechanical ventilation and drainage via chest tubes or video-assisted thoracoscopy surgery (VATS). Although there are no general guidelines for managing empyema specifically in children, studies have shown that primary VATS can result in shorter hospital stays compared to initial chest tube drainage followed by VATS [13].

Another dangerous sequela of an RPA is LS, which is defined as an oropharyngeal infection that leads to thrombophlebitis of the internal jugular vein and septicemia [14]. LS was first reported in the late 19th century, and the mortality rate associated with it has decreased significantly since then, primarily due to the use of antibiotics, now hovering around 4-18% [15]. However, in cases similar to ours, delayed diagnoses, antibiotic resistance, and multibacterial etiologies can make standard treatments complex. Additionally, the shock associated with septicemia can necessitate vasopressors via central venous access, which are associated with an increase in mortality and healthcare costs [16].

Our case highlights the unique anesthetic challenges in advanced cases of LS, particularly the need to manage a dynamic airway and coordinate multiple medications. Additionally, extubation decisions should extend beyond standard respiratory criteria to account for infection control and systemic illness. Effective sedative, analgesic, and paralytic drugs are critical components to consider for maintaining a balance between safety, comfort, and the ability to assess the patient neurologically. Dexmedetomidine is particularly useful in patients requiring mechanical ventilation because it causes minimal respiratory depression, facilitates easier weaning, promotes faster extubation, decreases sympathetic outflow from the locus coeruleus during postoperative recovery, and may reduce the need for higher doses of opioids and benzodiazepines [17]. Although opioid and benzodiazepine use should be limited due to safety concerns related to brain development in neonates, further research is needed to determine their impacts [18]. The specific antibiotics used to treat infections arising from the RPA in LS typically consist of clindamycin or vancomycin plus a third-generation cephalosporin, mainly to target Staphylococcus aureus or Streptococcus pyogenes [19]. However, our patient was found to be allergic to vancomycin a few days prior to admission, which added to a constantly changing antibiotic regimen, all failing to improve his status. As demonstrated in our case, these management challenges can surpass the capabilities of a given hospital system, necessitating transfer to a more advanced intensive care unit.

## Conclusions

This case shows the significant anesthetic and perioperative challenges involved in managing a complex RPA in an infant, further complicated by LS, bilateral empyemas, and a pseudoaneurysm of the internal carotid artery. Intubation was successfully achieved using video laryngoscopy while preserving spontaneous breathing, highlighting the value of a cautious, well-planned approach to airway management in the setting of deep neck infections. The spread of infection into the mediastinum and vascular structures also illustrates the importance of timely imaging, surgical drainage, and adjustments in antimicrobial treatment. For anesthesiologists, this case reinforces the importance of individualized sedation strategies, prolonged ventilatory support, and seamless collaboration with surgical and critical care teams to navigate airway instability and systemic illness. Ultimately, the patient’s recovery following coordinated, multidisciplinary care demonstrates the essential role of anesthesiology in managing life-threatening pediatric infections.
